# Smart bracelet to assess physical activity after cardiac surgery: A prospective study

**DOI:** 10.1371/journal.pone.0241368

**Published:** 2020-12-01

**Authors:** Marie Hauguel-Moreau, Cécile Naudin, Lee N’Guyen, Pierre Squara, Julien Rosencher, Serge Makowski, Fabrice Beverelli

**Affiliations:** 1 Cardiology Department, CMC Ambroise Paré, Neuilly sur Seine, France; 2 Research Department, CMC Ambroise Paré, Neuilly sur Seine, France; 3 Critical Care Department, CMC Ambroise Paré, Neuilly sur Seine, France; IRCCS Policlinico S.Donato, ITALY

## Abstract

**Objectives:**

Little is known about the physical activity of patients after cardiac surgery. This study was designed to assess this activity using a connected bracelet.

**Methods:**

In this prospective, monocentric study, patients scheduled for cardiac surgery were offered to wear an electronic bracelet. The main objective was to measure the physical activity recovery. Secondary objectives were the predictors of the correct use of the monitoring system, of the physical recovery and, if any, the relationship between physical activity and out-of-hospital morbidity.

**Results:**

One hundred patients were included. Most patients (86%) were interested in participating in the study. The compliance to the device and to the study protocol was good (94%). At discharge, the mean number of daily steps was 1454 ± 145 steps, increasing quite homogeneously, reaching 5801±1151 steps at Day 60. The best fit regression curve gave a maximum number of steps at 5897±119 (r^2^ = 0.97). The 85% level of activity was achieved at Day 30±3. No predictor of noncompliance was found. At discharge, age was independently associated with a lower number of daily steps (p <0.001). At Day 60, age, peripheral arterial disease and cardio-pulmonary bypass duration were independently associated with a lower number of daily steps (p = 0.039, p = 0.041 and p = 0.033, respectively).

**Conclusions:**

After cardiac surgery, wearing a smart bracelet recording daily steps is simple, well tolerated and suitable for measuring physical activity. Standard patients achieved around 6000 daily steps 2 months after discharge. 85% of this activity is reached in the first month.

**Clinical trial registry number:**

NCT03113565

## Introduction

Rehabilitation after cardiac surgery improves physical and psychological outcomes. A recent Cochrane review reported improved quality of life, reduced hospital readmissions and mortality [1]. Core components of cardiac rehabilitation include patient assessment, exercise training, diet counseling, risk factor control, patient education and psychosocial management with exercise training [2, 3]. In addition, promoting early training exercise, such as daily walking, may reduce muscle atrophy and recovery time [4, 5].

Hospital discharge is getting earlier and earlier after cardiac surgery [6]. Patient follow-up is a concern as major complications mainly occur within the first month. Decreased physical activity is an independent predictor for a complicated postoperative recovery in patients aged 65 years or older undergoing elective cardiac surgery [7].

The use of connected bracelets and smartwatches is increasing in medicine following significant improvements into both hardware and software components [8]. They seem to be well tolerated by the patients. A portable wearable wristband-type hand orthotic was rated overall satisfactory by stroke survivors [9].

This study was designed to measure the physical activity of patients after cardiac surgery using a connected bracelet and further, to see if this activity may be predicted by the perioperative status and/or may predict the occurrence of complications.

## Materials and methods

### Study population

BECSUP (Bracelet Electronique Connecté pour le SUivi des Patients après chirurgie cardiaque) was a monocentric prospective study conducted in patients scheduled for cardiac surgery in one teaching cardiothoracic hospital (Centre medico-chirurgical Ambroise Paré, Neuilly-sur-Seine, France). Patients older than 18 years old scheduled for cardiac surgery were screened for inclusion, regardless of the type of intervention. Non-inclusion criteria were pregnancy, refusal of consent, technical inability to use the device, inability to understand the protocol, and pre-existing disability that does not allow walking normally (not linked to cardiac pathology leading to surgery programmed). Since we looked to evaluate the number of daily steps usually performed by a standard patient, we excluded patients with in-hospital or extra-hospital serious adverse events. A cardiac rehabilitation program was proposed to all patients by the medical team. Patients who did not wear the bracelet for at least 30 days were secondarily excluded as well as patients with severe postoperative complications. All patients gave written informed consent. The study protocol was approved on January 2017 by an ethics committee (CPP Ile-De-France 7, Kremlin-Bicêtre) and published under n° NCT03113465 on clinicaltrials.gov. A patient representative was included in the research team. This was an exploratory study and it was decided to include 100 patients.

### Material

We used the *Withings Go*^®^ electronic bracelet as an activity tracker. It was worn on the patient’s wrist during the whole day, from discharge (Day 0) to Day 60. Following data were recorded: ID number, date, time, and number of steps per day. The bracelet was connected to an anonymous secured database. Data were hourly and automatically wireless transferred to the patient’s mobile phone (or tablet) and to a follow-up application (*CardioReport*^®^, Medireport, Paris).

### Procedure

All patients were encouraged to wear the electronic bracelet before hospital discharge. An application was downloaded on their smartphone (or tablet) by the study team whenever possible and checked for appropriate functioning. Clear explanations about the functioning of the bracelet were provided to patients by the research team.

Patient and hospitalization data were reported to an eCRF. Patients were contacted by phone call at Day 30 and Day 60 to collect pre-determined events (Table 3) and to answer a satisfaction questionnaire (Table 4). If no activity was detected on a patient device for more than 5 days, the patient was contacted to establish the reason for inactivity.

### Objectives

The main objective of the study was to measure the physical activity after cardiac surgery using the connected bracelet. The primary outcome was the patient’s number of daily steps. Secondary objectives were 1) to determine if different perioperative criteria were predictive of the correct use of the monitoring system, 2) to determine the predictors of a physical recovery in line with the goals and 3) to determine if out-of-hospital morbidity and mortality can be predicted from the collected information.

### Statistical analysis

Statistical analyses were performed using R software version 3.2.4 (The R Foundation for Statistical Computing; Vienna, Austria). Categorical variables were reported as numbers (percentages), and continuous variables as means ± SDs if normally distributed or medians (25th to 75th interquartile ranges) if not normally distributed. Categorical variables were compared using the chi-square test or Fisher exact test. Statistical significance was determined by a p-value of <0.05. A non-linear regression model was applied on daily steps per day to evaluate the maximum estimate days and when 85% of the maximal daily steps were achieved. Multivariable associations between predictors and daily steps were examined using a linear regression model. Selection of candidate variables for this model was based on clinical rationale.

## Results

### Study population

**Fig 1:**
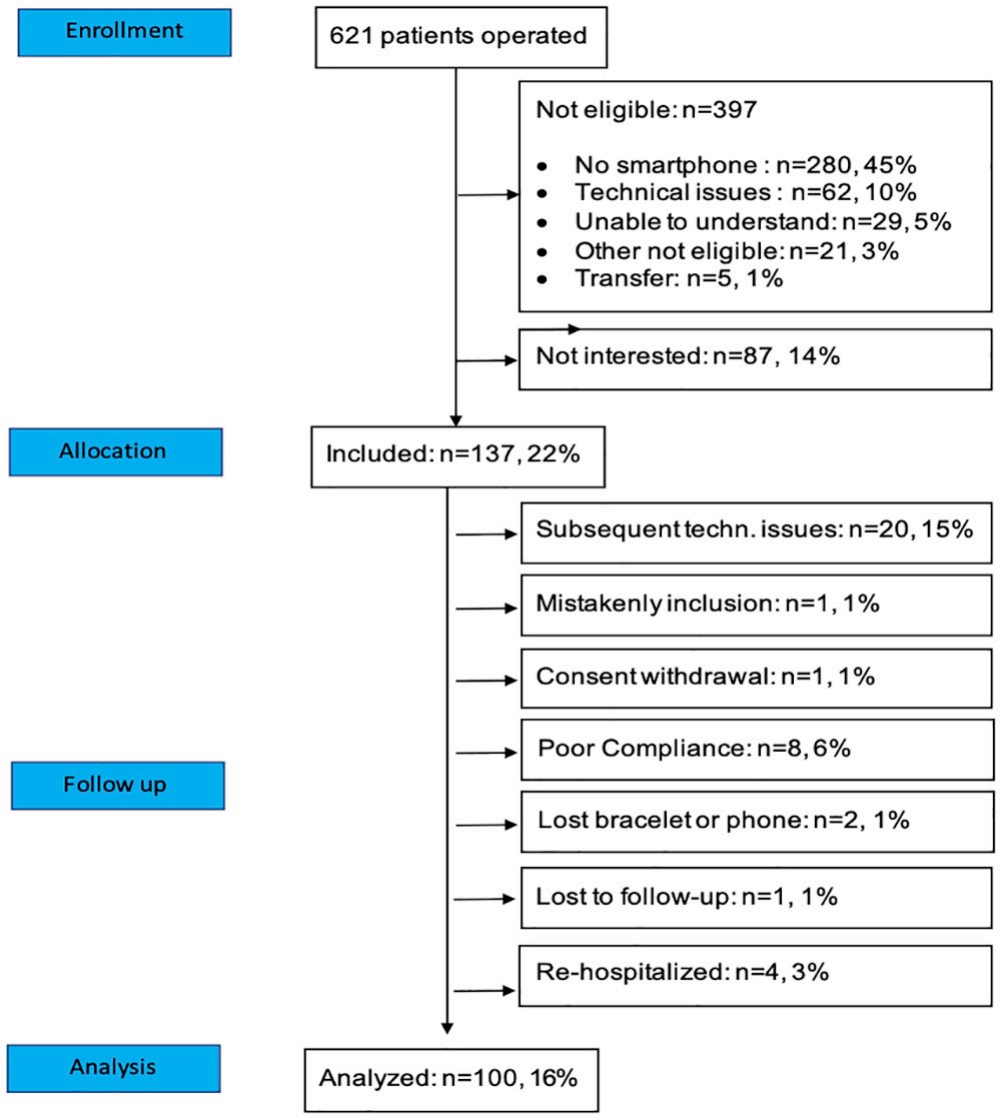
Study consort flow chart.

Between September 2017 and June 2018, 621 patients were admitted for scheduled cardiac surgery. After applying the non-inclusion criteria, 137 patients signed informed consent and 37 were secondarily excluded (see consort flow chart, Fig 1). Finally, 100 patients were analyzed. All of them followed at least one month of standard rehabilitation program in dedicated hospitals: 84 as residents and 16 as ambutatory patients.

The baseline characteristics of the study population are provided in Table 1. Patients were at standard risk of mortality and adverse events, with most having normal preoperative status and being discharged by Day 9 [8, 10] after intervention. There were no significant differences in these data between the analyzed patients (n = 100) and excluded patients (n = 37).

**Table 1:** Main patient perioperative characteristics.

	Study patients	Excluded	p
	n = 100	n = 37	
Age, (years)	63.8 [58.7–71.4]	63.0 [55.5–68.5]	NS
Gender, (F/M)	15/85	7/30	NS
BMI, (kg/m^2^)	26.6 [24.3–29.8]	26.6 [22.9–29.1]	NS
Angina:			
Stable angina, (%)	37	24	NS
ACS, (%)	8	14	NS
Hypertension, (%)	60	51	NS
Chronic lung disease, (%)	18	30	NS
Diabetes mellitus, (%)	16	30	NS
Smoker, (%)	58	49	NS
Peripheral vascular disease or stroke, (%)	6	11	NS
Preoperative LVEF, (%)	65 [60–70]	65 [57–68]	NS
Preoperative PA pressure, (mmHg)	30 [28–36]	33 [31–39]	NS
Preoperative creatinine, (μmol/l)	85 [75–100]	82 [73–95]	NS
Euroscore 2	0.8 [0.6–1.0]	0.8 [0.6–1.1]	NS
Surgery performed:			
Isolated CABG, (%)	54	43	NS
Isolated AVR, (%)	19	27	NS
Isolated MV repair, (%)	15	5	NS
Isolated MVR, (%)	2	8	NS
TV annuloplasty, (%)	0	3	NS
Aorta repair, (%)	1	0	NS
Cardiac myxoma resection, (%)	1	3	NS
CABG + AVR, (%)	4	0	NS
CABG + MV repair, (%)	0	3	NS
AVR + TV annuloplasty, (%)	1	0	NS
MV + TV annuloplasty, (%)	3	6	NS
CPB, (min)	77 [65–89]	65 [52–90]	NS
LOS, (days)	11 [10–13]	11 [10–14]	NS

BMI: Body Mass Index; LVEF: Left Ventricle Ejection Fraction; PA: Pulmonary Artery; CABG: Coronary Artery Bypass Graft; AVR: Aortic Valve Replacement; MV: Mitral Valve; MV: Mitral Valve Repair; TV: Tricuspid Valve; CPB: Cardio Pulmonary Bypass duration; LOS: Length of Stay. Values are reported as medians [IQRs].

### Daily steps

**Fig 2:**
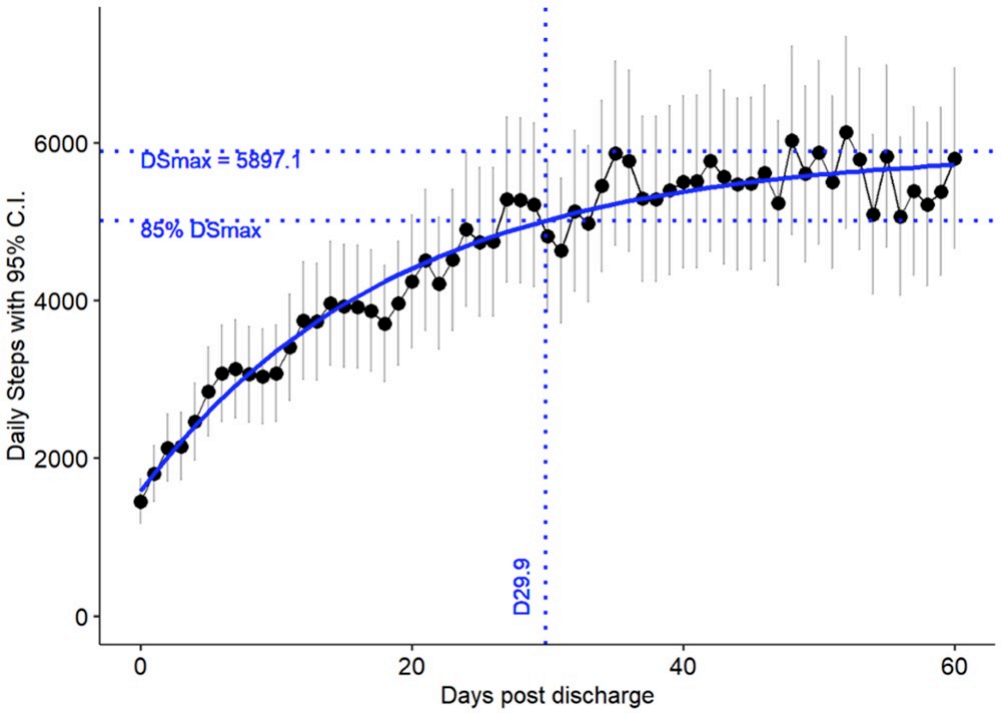
Daily steps performed after cardiac surgery. Black points = mean daily steps plus variability (±2SD) as shown by vertical lines. Blue curve = best fit regression, r^2^ = 0.97. DSmax: Extrapolated maximum of daily steps.

At discharge (Day 0 of the study), the mean number of daily steps was 1454±145 steps. This physical activity increased progressively with a quite homogeneous interpatient profile (Fig 2), reaching 5801±580 steps at Day 60. The inter-patient variability was moderate, with the 2SD highest activity less than 50% above the 2SD lowest activity. The best-fit regression curve showed a maximum daily number of steps at 5897±119 (r^2^ = 0.97). The 85% level of the maximum number of steps was achieved at 30 [27–33] days after discharge (Fig 2).

### Impact of perioperative factors on daily steps

Most patients (86%) were interested in participating in the study. The compliance to the device and to the study protocol was good (94%). No predictor of noncompliance was found due to the great proportion of technical issues as compared to noncompliance. At discharge, age was independently associated with a lower number of daily steps (p <0.001). At Day 60, age, peripheral arterial disease and cardio-pulmonary bypass duration were independently associated with a lower number of daily steps at Day 60 (p = 0.039, p = 0.041 and p = 0.033, respectively) (Table 2).

**Table 2:** Multivariate linear regression model predicting daily steps.

	Day 0			Day 60		
Predictors	Estimates	CI	p	Estimates	CI	p
(Intercept)	5293	2900–7685	<0.001	11708.80	1927.40–21490.20	0.021
Age (years)	-31	-52 –-10	0.005	-92.14	-178.30 –-5.97	0.039
Diabetes mellitus	-280	-871–310	0.355	497.74	-1919.55–2915.04	0.687
Smoker	60	-360–481	0.778	267.38	-1453.80–1988.57	0.761
Peripheral vascular disease or stroke	-419	-1305–466	0.355	-3822	-7445 –-199	0.041
Preoperative LVEF (%)	-21	-47–4	0.101	63	-41–167	0.246
CPB (min.)	-5.2	-13–2.7	0.199	-36	-67 –-3.0	0.033

### Safety and satisfaction

As predicted by the Euroscore and considering the exclusion of patients having severe adverse events, the study mortality was very low (no death and one loss of follow-up). Morbidity was also low with 3% prolonged hospitalization and 6% rehospitalization for any reason (Table 3). Therefore, out-of-hospital morbidity and mortality were not predicted from the collected data. The results of the questionnaire at Day 30 and 60 are given in Table 4. At Day 60, physical activity was limited by dyspnea (as compared to preoperative status) in 7% of the patients and by pain in 4% of the patients. The connected bracelet was seen as useful; 61% of patients considered that it has influenced their rehabilitation and 41% that it changed their way of life. The connected bracelet and process to transfer the information was acceptable; 77% of the patients decided to continue wearing it and only 4% considered it as a constraint.

**Table 3:** Extra-hospital events at Day 30 and Day 60. Y = yes, N = no, and DN = don’t know.

	DAY 30			DAY 60		
Complication occurrence (Y/N/DN)	17	79	4	16	80	4
-Major complications (Y/N/DN)	5			1		
-Minor complications (Y/N/DN)	12			15		
Related to cardiac surgery (Y/N/DN)	14	3	-	9	5	2
Prolonged hospitalization (Y/N/DN)	3	91	6	-	-	-
Rehospitalization (Y/N/DN)	5	92	3	3	93	4
Death (Y/N/DN)	-	99	1	-	99	1

**Table 4:** Patient questionnaire at Day 30 and Day 60. Y = yes, N = no, and DN = don’t know.

	DAY 30			DAY 60		
How do you score your actual physical activity? (better/same/less)	62	7	26	73	16	7
How do you score your actual breathing? (better/same/less)	60	20	13	71	18	7
Have you experienced scar pain? (Y/N/DN)	46	48	6	36	60	4
If yes, is this pain limiting your activity? (Y/N/DN)	13	33	-	7	29	-
Do you believe that the bracelet influenced your rehabilitation? (Y/N/DN)	53	38	9	61	32	7
Is the bracelet representing a constraint in your daily life? (Y/N/DN)	-	94	6	7	89	4
Is the bracelet changing your way of life? (Y/N/DN)	23	71	6	41	55	4
Would you accept pursuing your monitoring using this bracelet? (Y/N/DN)	83	11	6	77	19	4
Do you believe that other connected devices would do better? (Y/N/DN)	64	19	17	69	20	11

## Discussion

This study shows that a connected bracelet is easy to use and well accepted by most patients as well as providing a few constraints. Half of them considered that this tool influenced their rehabilitation. The most important limitation for using the connected bracelet was technical. Our results suggest that postcardiac surgery patients achieved around 6000 daily steps 2 months after discharge. The increase in the daily steps average is quite homogeneous, following a quite common curve and reaching 85% of the maximum activity near one month after discharge.

As previously reported, age was a constant independent factor affecting the recovery of patients [10]. Peripheral arterial disease, including past stroke, also affected the maximum activity steps that a patient could perform. Cardiopulmonary bypass duration was the last independent factor affecting the number of daily steps. This is not surprising since cardiopulmonary bypass duration is a global indicator of surgical complexity and known to be an independent predictive factor of outcome after cardiac surgery [11]. There were not enough patients to determine if a specific intervention could affect the number of steps per day during recovery.

To our knowledge, this study was the first that measures the recovery profile and the level of activity as assessed by daily steps after standard cardiac surgery. Therefore, few data in the literature can be compared to our results [12]. Activity trackers may contribute to better treatments and more positive outcomes [13]. It has also been shown that physical activity assessed by motion sensors in patients with heart failure was linked with exercise capacity and was predictive of the disease severity [14, 15]. Wearing an activity tracker has the potential to increase physical activity participation [16, 17]. We can also speculate that monitoring patient activity will also enable to identify patients at risk for complications or requiring enhanced follow-up [18]. Thus, when better standardization will allow a majority of patients to have access to connected devices, they could become a standard of postoperative follow-up. They could act to motivate the patients (self-coaching). Using the basic system of this study, 60% of the patients considered that it improved their recovery. We can speculate that more sophisticated software will help a majority of patients. Connected bracelet could also help clinicians to target the appropriate daily physical activity, adapted to the specific situation of the patient, and finally provide an overview of the progress of the recovery. We found three independent factors affecting the number of daily steps. Further studies may better indicate which type of patients and/or which patient characteristics may benefit from reinforced protocols. This would promote appropriate interventions either to shorten the rehabilitation or to strengthen it.

### Limitations

A lot of patients were noneligible. From a cohort of 621 patients, only 100 were analyzed. However, 297 patients were unable to participate, mostly due to neurological, psychological or motor limitations. Therefore, a generalization of our results is only suitable for the 224 patients eligible for the study. Technical problems were the main reason for reducing this number to 100. Indeed, a specific recent Android or Apple operating system phone was needed to connect the smart bracelet to the mobile application. The daily wear of a bracelet was the second reason for non-inclusion. In the absence of immediate benefit, 15% of our patients were not interested in wearing a bracelet. Further studies are needed to demonstrate that daily monitoring of patients with a smart device can anticipate adverse events or their severity and to convince the patient of its interest. We assume that this technological selection did not generate different results than a random selection would have given. The number of daily steps performed by our patients before surgery was not measured, since this study was not designed to compare the number of daily steps performed before and after cardiac surgery. However, preoperative walking scores would have been impacted by the heart disease. None of the patients included had a physical disability that prevented them from walking and being able to perform standard exercise. Overall, 62% of the patients at day 30 and 73% at day 60 considered that their physical activity was improved.

Wearable activity trackers have become popular for assessing the daily physical activity of individuals. However, the validity of these devices in step counts has raised concerns [19] and debate [20]. Pedometers generated significant errors at slow speeds, slower than 0.6 m/s (2.16 km/h or 1.24 mph) [21, 22]. A recent study compared the ability of ten different tracker activity systems for measuring steps. Although considerable inter-device variability was found, the *Withings* system that we used was one of the two providing the most accurate measures under three different conditions (i.e., treadmill, over-ground, and 24-hour conditions) [23].

## Conclusion

After cardiac surgery, wearing a smart bracelet recording daily steps is simple, well tolerated and useful to measure physical activity recovery. Standard postcardiac surgery patients achieved around 3000–5000 daily steps one month after discharge and reached a plateau of around 5000–7000 steps at two months after discharge. Only 45% of the patients able to participate, benefited from this technology, most often for technological reasons. Improving the standardization of connected devices will help to enlarge the proportion of patient candidates for this type of monitoring.

## Supporting information

S1 File TREND statement checklist. (PDF)

S2 File (PDF)

S3 File (PDF)

S4 File (PDF)
