# Supplementary material for: Smart bracelet to assess physical activity after cardiac surgery: A prospective study
Source: PLoS One. 2020 Dec 1;15(12):e0241368. doi: 10.1371/journal.pone.0241368 (PMC7707519; doi:10.1371/journal.pone.0241368)
Supplement: S2 File — (PDF) [file pone.0241368.s002.pdf]

ClinicalTrials.gov Protocol Registration and Results System (PRS) Receipt
Release Date: May 17, 2019

ClinicalTrials.gov ID: NCT03113565

Study Identification

Unique Protocol ID: 2016/02

Brief Title: Connected Electronic Wrist Strap for Patient Follow-up After Cardiac Surgery (BECSuP)

Official Title: Connected Electronic Wrist Strap for Patient Follow-up After Cardiac Surgery

Secondary IDs:

Study Status

Record Verification: May 2019

Overall Status: Completed

Study Start: August 9, 2017 [Actual]

Primary Completion: June 10, 2018 [Actual]

Study Completion: June 10, 2018 [Actual]

Sponsor/Collaborators

Sponsor: CMC Ambroise Paré

Responsible Party: Sponsor

Collaborators:

Oversight

U.S. FDA-regulated Drug: No

U.S. FDA-regulated Device: No

U.S. FDA IND/IDE: No

Human Subjects Review: Board Status: Approved
Approval Number: SC 16-034
Board Name: CPP Ile de France 7
Board Affiliation: Hopital de Bicêtre
Phone: 0145212846
Email: cpp.idf.7-bicetre@orange.fr
Address:

CPP ile de France 7
Hopital de Bicêtre
78, rue du Général Leclerc
94270, Le Kremlin-Bicêtre Cedex

Data Monitoring: No

FDA Regulated Intervention: No

Study Description

Brief Summary: After cardiac surgery, patients' follow-up after discharge is a major public health issue. Since the main complications occur mostly during the first extra-hospital month, a follow-up period becomes necessary as the average duration of hospitalization tends to decrease. The resumption of normal physical activity is rarely transmitted and when complications arise, the healthcare team is most often informed late.

An electronic wristband is worn by the patient during the day, between the day of discharge from the hospital (D0) and the end of the second extra-hospital month (D60). The data recorded by the wristband include : bracelet ID, date, time and number of steps per day.

The primary objective of the study is to measure the resumption of physical activity after elective cardiac surgery. This objective will be quantified by the number of daily footsteps.

A secondary objective is to determine perioperative predictors of the physical resumption.

Detailed Description:

Conditions

Conditions: Cardiac Surgery
Surgery--Complications
Cardiac Disease
Physical Disability

Keywords:

Study Design

Study Type: Observational [Patient Registry]

Observational Study Model: Cohort

Time Perspective: Prospective

Biospecimen Retention:

Biospecimen Description:

Enrollment: 137 [Actual]

Number of Groups/Cohorts: 1

Target Follow-Up Duration: 2 Months

Groups and Interventions

Intervention Details:

Behavioral: Count daily number of footsteps.

Using a connected electronic wristband to mainly quantify daily number of footsteps.

Outcome Measures

Primary Outcome Measure:

1. Measure the resumption of physical activity after cardiac surgery by counting the number of daily footsteps. Count the number of daily footsteps

   [Time Frame: 2 months]

Eligibility

Study Population: Adult patients who underwent elective cardiac surgery, regardless of indication and type of intervention.

Sampling Method: Non-Probability Sample

Minimum Age: 18 Years

Maximum Age:

Sex: All

Gender Based:

Accepts Healthy Volunteers: No

Criteria: Inclusion Criteria:

- Adult patients who underwent elective cardiac surgery, regardless of indication and type of intervention.

Exclusion Criteria:

- Refuse of the patient,
- Misunderstanding of the system (electronic wristband, application) or the principle of the study (language problem, cerebral vascular sequelae),
- Pre-existing handicap that does not allow walking (not related to the cardiac pathology leading to the planned cardiac surgery).
- Patients who are unable to understand the content of the information delivered
- Pregnant women can not be included in the study.

Contacts/Locations

Central Contact Person: FABRICE BEVERELLI, MD
Telephone: 0033146418777
Email: beverelli@free.fr

Central Contact Backup:

Study Officials:

Locations: **France**
CMC AMBROISE PARE
Neuilly-sur-Seine, ILE DE France, France, 92200
Contact: Marie-Caroline Mérand marie-caroline.merand@clinique-a-pare.fr
Principal Investigator: Fabrice Beverelli, MD
Sub-Investigator: Serge Makowski, MD
Sub-Investigator: Alain Brusset, MD
Sub-Investigator: Philippe Estagnasié, MD
Sub-Investigator: Jean-Claude Dib, MD
Sub-Investigator: Olivier Bélliard, MD
Sub-Investigator: Lee S Nguyen, MD,MSc

Sub-Investigator: Pierre Squara, MD

IPDSharing

Plan to Share IPD: No

References

Citations:

[Study Results] Ball L, Costantino F, Pelosi P. Postoperative complications of patients undergoing cardiac surgery. Curr Opin Crit Care. 2016 Aug;22(4):386-92. doi: 10.1097/MCC.0000000000000319. Review. PubMed 27309972

[Study Results] Kim DH, Kim CA, Placide S, Lipsitz LA, Marcantonio ER. Preoperative Frailty Assessment and Outcomes at 6 Months or Later in Older Adults Undergoing Cardiac Surgical Procedures: A Systematic Review. Ann Intern Med. 2016 Nov 1;165(9):650-660. doi: 10.7326/M16-0652. Epub 2016 Aug 23. Review. PubMed 27548070

[Study Results] Mazzeffi M, Zivot J, Buchman T, Halkos M. In-hospital mortality after cardiac surgery: patient characteristics, timing, and association with postoperative length of intensive care unit and hospital stay. Ann Thorac Surg. 2014 Apr;97(4):1220-5. doi: 10.1016/j.athoracsur.2013.10.040. Epub 2013 Dec 21. PubMed 24360878

[Study Results] Hulzebos EH, Smit Y, Helders PP, van Meeteren NL. Preoperative physical therapy for elective cardiac surgery patients. Cochrane Database Syst Rev. 2012 Nov 14;11:CD010118. doi: 10.1002/14651858.CD010118.pub2. Review. PubMed 23152283

[Study Results] Mainini C, Rebelo PF, Bardelli R, Kopliku B, Tenconi S, Costi S, Tedeschi C, Fugazzaro S. Perioperative physical exercise interventions for patients undergoing lung cancer surgery: What is the evidence? SAGE Open Med. 2016 Oct 19;4:2050312116673855. eCollection 2016. Review. PubMed 27803808

Links:

Available IPD/Information:
