# Supplementary material for: Smart bracelet to assess physical activity after cardiac surgery: A prospective study
Source: PLoS One. 2020 Dec 1;15(12):e0241368. doi: 10.1371/journal.pone.0241368 (PMC7707519; doi:10.1371/journal.pone.0241368)
Supplement: S3 File — (PDF) [file pone.0241368.s003.pdf]

Summary

Full title	Smart bracelet to assess physical activity after cardiac surgery
Short title	BECSUP
Version	Novembre 1, 2016
Reference	2016/02
N° RCB	2016-A01673-48
Promotion	CMC Ambroise Paré
Investigator	Dr Fabrice Beverelli
Scientifique manager	Dr Pierre Squara

Background	The monitoring of cardiac surgery patients after discharge is an issue since the main complications occur during the first month. A follow-up period becomes necessary especially because the length of hospitalization tends to decrease. Return to normal physical activity is only rarely transmitted to the healthcare team and when the complications occur, the healthcare team is the most often informed late. Today, tools for simple assessment of physical activity are easily available but thay are used by patients heuristically.
Main objective	The main objective of the study is to measure the recovery of a physical activity after scheduled cardiac surgery by using a connected electronic bracelet. This objective will be quantified by the number of daily steps.
Secondary objectives	1. To determine if there is a link between the use of the bracelet and pre-, per- and post-operative criteria (appendix 1). 2. To determine the pre-, per- and early postoperative criteria (hospital phase) predictive of resumption of physical activity conforms to the usual objectives set for the patient before discharge of the establishment.<br>3. To determine if the morbi - extra-hospital mortality (appendix 3-4) can be predicted by the monitoring of the physical activity.
Protocol	The The Withings Go electronic bracelet (see appendix 5) is worn by the patient on his wrist, at least from sunrise to bedtime, between the day of discharge from the hospital (D0) and the end of second extra-hospital month (D60). Recorded data: bracelet ID, date / time, number of steps taken per day, and heart rate averaged by 5 minutes. The connected bracelet is connected to a anonymous cloud database allowing only the identification of the bracelet. The unique identification will be recorded and linked to the patient file. The data is automatically transferred every hour by through an application on the mobile phone or a tablet connected to the internet, for the duration of the study that is to say the exit from the hospital sector (D0) until the 60th day (D60).
Study population	All adult patients who admitted in our institution for scheduled cardiac surgery, regardless of indication and of the type of intervention.
Inclusion criteria	All adult patients who admitted in our institution for scheduled cardiac surgery, regardless of indication and of the type of intervention.

Non-inclusion criteria	Patient refusal ; poor understanding of the system (electronic bracelet, application), poor undestanding of the principle of the study (language problem, cerebrovascular sequelae), pre-existing handicap that does not allow walking (not linked to cardiac pathology leading to cardiac surgery programmed); patients unable to understand the content of information provided and pregnant women do cannot be included in the study.
Exclusion criteria	Patients not adhering to the protocol, refusing to wear the bracelet in the first month. These patients will be replaced in the study if the exclusion occurs before D30.
New product	NA
Comparator	NA
Acts added by the protocol	1) wearing a connected bracelet; 2) accepting a follow-up on D30 and D60 with a phone call to collect extra-hospital events; 3) answering a quality of life questionnaire (cf. appendix 4) either via the application or via the telephone call.
Risk added by the protocol	None
Benefit added by the protocol	No individual benefit can be advanced in this study, No individual benefit can be advanced in this study, however patient involvement in the study is likely to trigger increased motivation for retrun to normal physical activity.
Expected interest	Determination of the predictive factors of post operative retunr to normal activity after cardiac surgery. Analysis of the links between resumption of physical activity and the level of morbidity and mortality outside the hospital. Assessment of the patient's quality of life post-cardiac surgery depending on the level of recovery activity.
Practical procedure	Before discharge from the hospital, all patients will be offered to wear an electronic bracelet for measuring the physical activity. For those who accept, the team in charge of the study will install an application on his/her mobile phone, laptop or tablet. An investigator will explain to the patient where and how to wear the device. The electronic bracelet is worn by the patient on his wrist during the day, from sunrise to bedtime, between the day of discharge from the hospital sector (D0) and the end of the second month extrahospital (J60). Data is transferred every hours through a phone app laptop or tablet connected to the internet, during the whole duration of the study, i.e. leaving the hospital sector (D0) until the 60th day (D60). Hospitalization data are retrieved and directly transferred to an eCRF. A phone call at D30 and D60 will detect the occurrence of events (see Annex 2) and respond to questionnaires (see appendices 3 and 4).
Numbrer of inclusions	100 patients will be included
Numbrer of center	This is a monocentric study
Duration	Inclusions : 10 months, follow up : 60 jdays after inclusion, then total duration: 12 months
Inclusions per month	It is planned to include 10 patients per month
