# Supplementary material for: Smart bracelet to assess physical activity after cardiac surgery: A prospective study
Source: PLoS One. 2020 Dec 1;15(12):e0241368. doi: 10.1371/journal.pone.0241368 (PMC7707519; doi:10.1371/journal.pone.0241368)

**Titre : Bracelet électronique connecté pour le suivi des patients après chirurgie cardiaque**
**Titre abrégé : « BECSuP »**
**Etude monocentrique**

# PROTOCOLE DE RECHERCHE DE SOINS COURANTS

## INFORMATIONS GENERALES

### REFERENCES DU PROTOCOLE

Numéro de code du protocole attribué par le gestionnaire : 2016/02
N° RCB : 2016-A01673-48
Numéro de version : version 1 du 7/11/2016

### GESTIONNAIRE DE L'ETUDE

CMC Ambroise Paré
25-27 boulevard Victor Hugo 92200 Neuilly sur Seine
Personne à contacter Marie-Caroline Mérand
Tel : 01 46 41 89 56, fax : 01 46 41 89 81
Courriel : marie-caroline.merand@clinique-a-par.fr

### INVESTIGATEUR COORDONNATEUR

Dr Beverelli Fabrice
Tel : 01 46 41 87 77, fax : 01 46 41 89 81
Courriel : beverelli@free.fr

### RESPONSABLE SCIENTIFIQUE

Dr Pierre SQUARA
Tel : 01 46 41 89 71, fax : 01 46 41 89 81
Courriel : pierre.squara@orange.fr

### Coordonnées des lieux de recherche

Dr Fabrice Beverelli
CMC Ambroise Paré
25-27 boulevard Victor Hugo
92200 Neuilly sur Seine

## SOMMAIRE

1 RÉSUMÉ SYNOPTIQUE ........................................................................................................ 5

2. JUSTIFICATION SCIENTIFIQUE ET DESCRIPTION GENERALE DE LA RECHERCHE 8

2.1 RESUME DES BENEFICES, LE CAS ECHEANT, ET DES RISQUES PREVISIBLES ET CONNUS POUR LES PERSONNES SE PRETANT A LA RECHERCHE ........................................................................................................ 8

2.2 CONFORMITÉ DE LA RECHERCHE ........................................................................................................ 8

2.3 DESCRIPTION DE LA POPULATION A ETUDIER ........................................................................................................ 8

2.4 REFERENCES A LA LITTERATURE SCIENTIFIQUE ET AUX DONNEES PERTINENTES SERVANT DE REFERENCE POUR LA RECHERCHE ........................................................................................................ 8

3. OBJECTIFS DE LA RECHERCHE, CRITERES EN RAPPORT AVEC CES OBJECTIFS ....... 9

3.1. OBJECTIF PRINCIPAL : MESURE ........................................................................................................ 9

3.2. OBJECTIFS SECONDAIRES ........................................................................................................ 9

3.2.1. OBJECTIF SECONDAIRE : FACTEURS EN RAPPORT AVEC L'UTILISATION ........................................................................................................ 9

3.2.2 OBJECTIF SECONDAIRE 2 : FACTEUR PRÉDICTIFS DE LA REPRISE D'ACTIVITÉ ........................................................................................................ 9

3.2.3 OBJECTIF SECONDAIRE 3 : LIEN AVEC LA MORBI–MORTALITÉ ........................................................................................................ 9

4. CONCEPTION ET DEROULEMENT DE LA RECHERCHE ........................................................................................................ 9

4.1. PLAN EXPERIMENTAL ........................................................................................................ 9

4.2. IDENTIFICATION DES SUJETS ........................................................................................................ 9

4.3. DEROULEMENT DE LA RECHERCHE ........................................................................................................ 9

4.4. TABLEAU RECAPITULATIF DE LA CHRONOLOGIE DE LA RECHERCHE ........................................................................................................ 10

4.5. DESCRIPTION DES TECHNIQUES UTILISEES ........................................................................................................ 11

4.6. DUREE PREVUE DE PARTICIPATION DES PERSONNES, DESCRIPTION DE LA CHRONOLOGIE ET DE LA DUREE DE LA RECHERCHE ........................................................................................................ 11

4.7. DESCRIPTION DES MESURES PRISES POUR REDUIRE ET EVITER LES BIAIS ........................................................................................................ 11

4.8. DESCRIPTION DES REGLES D'ARRET DEFINITIF OU TEMPORAIRE ........................................................................................................ 11

4.9. IDENTIFICATION DE TOUTES LES DONNEES A RECUEILLIR DIRECTEMENT DANS LES CAHIERS D'OBSERVATION, QUI SERONT CONSIDEREES COMME DES DONNEES SOURCE ........................................................................................................ 12

5. POPULATION ETUDIEE ........................................................................................................ 12

5.1. DESCRIPTION DE LA POPULATION A ETUDIER ........................................................................................................ 12

5.2. CRITERES D'INCLUSION ........................................................................................................ 12

5.3. CRITERES DE NON-INCLUSION .......... 12
5.4. CRITERES D'EXCLUSION ET MODALITES .......... 13

6. EVALUATION DE LA SECURITE .......... 13

7. STATISTIQUES .......... 13

7.1. DESCRIPTION DES METHODES STATISTIQUES PREVUES .......... 13
7.2. NOMBRE PREVU DE PERSONNES A INCLURE DANS LA RECHERCHE AVEC SA JUSTIFICATION STATISTIQUE .......... 13
7.3. DEGRE DE SIGNIFICATION PREVU .......... 14
7.4. CRITERES STATISTIQUES D'ARRET DE LA RECHERCHE .......... 14
7.5. METHODE DE PRISE EN COMPTE DES DONNEES MANQUANTES, INUTILISEES OU NON VALIDES .... 14
7.6. GESTION DES MODIFICATIONS APPORTEES AU PLAN STATISTIQUE INITIAL .......... 14
7.7. CHOIX DES PERSONNES A INCLURE DANS LES ANALYSES .......... 14

8. DROIT D'ACCES AUX DONNEES ET DOCUMENTS SOURCE .......... 14

8.1. ACCES AUX DONNEES .......... 14
8.2. DOCUMENTS SOURCE .......... 15
8.3. CONFIDENTIALITE DES DONNEES .......... 15
8.4. CAHIER D'OBSERVATION ET RECUEIL DES DONNEES .......... 15

9. CONTROLE ET ASSURANCE DE LA QUALITE .......... 16

9.1. CONTRÔLE QUALITE DES DONNEES .......... 16
9.2. AUDIT/INSPECTION .......... 16

10 CONSIDERATIONS ETHIQUES .......... 16

10.1. JUSTIFICATION DE LA TYPOLOGIE DE LA RECHERCHE EN SOINS COURANTS .......... 16
10.2. DEMARCHES REGLEMENTAIRES PREALABLES A LA MISE EN ŒUVRE DE LA RECHERCHE .......... 16

11. TRAITEMENT DES DONNEES ET CONSERVATION DES DOCUMENTS ET DES DONNEES RELATIVES A LA RECHERCHE .......... 17

12. FINANCEMENT ET ASSURANCE .......... 17

FINANCEMENT DE L'ETUDE .......... 17

ASSURANCE .......... 17

13. REGLES RELATIVES A LA PUBLICATION .......... 17

14. REFERENCE .......... 18

15. ANNEXES .......... 18

## 1 RÉSUMÉ SYNOPTIQUE

| | |
|---|---|
| Titre complet | Bracelet électronique connecté pour le suivi des patients après chirurgie cardiaque |
| Titre abrégé | BECSUP |
| Version | 1 du 7 novembre 2016 |
| Référence gestionnaires | 2016/02 |
| N° RCB | 2016-A01673-48 |
| Gestionnaire | CMC Ambroise Paré |
| Investigateur coordonnateur | Dr Fabrice Beverelli |
| Responsable scientifique | Dr Pierre Squara |

| | |
|---|---|
| Justification scientifique | Pendant la période post-opératoire de chirurgie cardiaque, le suivi des patients après leur retour à domicile est un enjeu important de santé publique car les principales complications surviennent essentiellement durant le premier mois extra-hospitalier. Une période de suivi devient d'autant plus nécessaire que la durée moyenne d'hospitalisation tend à diminuer. Ainsi, la reprise d'une activité physique normale n'est que rarement transmises à l'équipe soignante. Lorsque les complications surviennent, l'équipe soignante est le plus souvent informée tardivement. Aujourd'hui, des outils de mesure simple de l'activité physique, disponibles dans le commerce, sont utilisés par les patients de manière aléatoire et/ou heuristique. |
| Objectif et critère d'évaluation principal | L'objectif principal de l'étude est de mesurer la reprise d'une activité physique après une chirurgie cardiaque programmée grâce à l'utilisation d'un bracelet électronique connecté. Cet objectif sera quantifié par le nombre de pas journaliers. |
| Objectifs et critères d'évaluation secondaires | 1. Déterminer s'il existe un lien entre l'utilisation du bracelet et des critères pré-, per- et post-opératoires (annexe 1).<br>2. Déterminer les critères pré-, per- et post-opératoires précoces (phase hospitalière) prédictifs d'une reprise d'activité physique conforme aux objectifs habituels fixés au patient avant sa sortie de l'établissement.<br>3. Déterminer si la morbi−mortalité extra−hospitalière (annexe 3-4) peut être prédite à à partir des données de surveillance de l'activité physique ainsi enregistrée. |
| Schéma expérimental | Le bracelet électronique Withings Go (cf. annexe 5) est porté par le patient à son poignet, au minimum du lever au coucher, entre le jour de la sortie du secteur hospitalier (J0) et la fin du deuxième mois extrahospitalier (J60). Les données enregistrées par le bracelet incluent : l'ID du bracelet, la date/heure, le nombre de pas effectué par journée, et la fréquence cardiaque moyennée par 5 minutes. Le bracelet connecté est relié à une base anonyme de données cloud permettant uniquement l'identification du bracelet. L'identification unique sera enregistrée et liée au dossier patient. Les données sont transférées automatiquement toutes les heures par l'intermédiaire d'une application sur le téléphone portable |

| | |
|---|---|
| | ou une tablette connectée à internet, pendant toute la durée de l'étude c'est-à-dire de la sortie du secteur hospitalier (J0) jusqu'au 60$^{ème}$ jour (J60). |
| Population concernée | Tous les patients adultes en capacité qui auront bénéficié d'une chirurgie cardiaque programmée, quelle que soit l'indication et le type d'intervention. |
| Critères d'inclusion | Les patients adultes ayant bénéficié d'une chirurgie cardiaque programmée, quelle que soit l'indication et le type d'intervention. |
| Critères de non inclusion | Refus du patient, mauvaise compréhension du système (bracelet électronique, application) ou du principe de l'étude (problème de langue, séquelles vasculaires cérébrales), handicap préexistant ne permettant pas la marche (non lié à la pathologie cardiaque ayant conduit à la chirurgie cardiaque programmée). Les patients en incapacité de comprendre le contenu des informations délivrées et les femmes enceintes ne pourront pas être inclus dans l'étude. |
| Critères d'exclusion | Les patients n'adhérant pas au protocole, refusant de porter le bracelet dans le premier mois. Ces patients seront remplacés dans l'étude si l'exclusion intervient avant J30. |
| Produits expérimentaux | NA |
| Produit de référence / comparateur | NA |
| Autres actes ajoutés par la recherche | Le port d'un bracelet électronique permettant la mesure de l'activité physique.<br>Un suivi à J30 et J60 par un appel téléphonique pour recueillir les événements extra hospitaliers ainsi que l'envoi d'un questionnaire sur le Smartphone des participants (cf. annexe 3) auquel s'ajoute à J60 le questionnaire qualité de vie (cf. annexe 4) soit via l'application soit via l'appel téléphonique . |
| Risques ajoutés par la recherche | Aucun risque n'est envisagé dans cette recherche |
| Bénéfices attendus pour les patients | Aucun bénéfice individuel ne peut être avancé dans cette étude, cependant une implication des patients dans l'étude est susceptible de déclencher une motivation accrue quant à la reprise d'une activité. |
| Retombées attendus | La détermination des facteurs prédictifs de reprise d'activité après une chirurgie cardiaque. Une analyse des liens entre la reprise d'activité physique et le niveau de la morbi-mortalité extra-hospitalière. L'évaluation de la qualité de vie du patient post-chirurgie cardiaque en fonction du niveau de reprise d'activité. |
| Déroulement pratique | Avant la sortie du secteur hospitalier, tous les patients se verront proposer de porter un bracelet électronique de mesure d'activité physique. Pour ceux qui accepteront, l'équipe en charge de l'étude installera une application sur le téléphone portable ou la tablette de chaque patient. Un investigateur expliquera au patient où et comment porter le dispositif. Le bracelet électronique est porté par le patient à son poignet pendant la journée, du lever au coucher, entre le jour de la sortie du secteur hospitalier (J0) et la fin du deuxième mois extrahospitalier (J60). Les données sont transférées toutes les heures par l'intermédiaire d'une application sur téléphone portable |

| | |
|---|---|
| | ou une tablette connectée à internet, pendant toute la durée de l'étude c'est-à-dire de la sortie du secteur hospitalier (J0) jusqu'au 60ème jour (J60). Les données d'hospitalisation sont récupérées et directement reportées dans un eCRF. Un appel téléphonique à J30 et à J60 permettra de détecter la survenue d'évènements (cf. annexe 2) et de répondre aux questionnaires (cf. annexes 3 et 4). |
| Nombre de sujets sélectionnés | Dans cette étude il est prévu d'inclure 100 patients |
| Nombre de centres | Cette étude est monocentrique, nationale. |
| Durée de la recherche | durée d'inclusion : 10 mois<br>durée de participation (traitement + suivi) : 60 jours après la sortie hospitalière<br>durée totale : 12 mois |
| Nombre d'inclusions prévues par centre et par mois | Il est prévu d'inclure 10 patients par mois |
| Analyse statistique | Il s'agit d'une étude exploratoire ; ce qui rend difficile le calcul du nombre de sujets à inclure.<br>En se basant sur les capacités de recrutement, l'objectif de conclure rapidement quant à l'intérêt de la méthode de manière à pouvoir, le cas échéant, mener une étude comparative, et la nécessité de disposer d'une puissance suffisante. Un échatillon de 100 patients permet, si une proportion est comprise entre 0.1 et 0.5 d'obtenir des limites de confiance à 95 % d'une taille raisonnable. On approchera donc le nomre de patients nécessair pour obtenir 100 consentements de participation. |
| Source de financement | Le financement de l'étude est assuré par le CMC Ambroise Paré |
| Comité de Surveillance Indépendant prévu | Oui/Non<br>Le gestionnaire n'a pas jugé nécessaire de mettre en place un comité de surveillance indépendant dans cette étude. |

## 2. JUSTIFICATION SCIENTIFIQUE ET DESCRIPTION GENERALE DE LA RECHERCHE

Pendant la période post-opératoire de chirurgie cardiaque, le suivi des patients après leur retour à domicile est un enjeu important de santé publique car les principales complications surviennent essentiellement durant le premier mois extra-hospitalier[1, 2]. Une période de suivi devient d'autant plus nécessaire que la durée moyenne d'hospitalisation tend à diminuer [3]. Ainsi, la reprise d'une activité physique normale n'est que rarement transmises à l'équipe soignante. Lorsque les complications surviennent, l'équipe soignante est le plus souvent informée tardivement. Aujourd'hui, des outils de mesure simple de l'activité physique [4,5], disponibles dans le commerce, sont utilisés par les patients de manière aléatoire et/ou heuristique.

L'étude que nous proposons a pour but de mesurer systématiquement l'activité physique après chirurgie cardiaque programmée grâce à l'utilisation d'un bracelet électronique connecté, par ailleurs déjà à la disposition du public à cet usage. Les facteurs prédictifs de la reprise d'activité physique seront aussi étudiés.

Une telle étude permettra d'estimer les paramètres d'une éventuelle deuxième étude visant à évaluer l'intérêt de la stimulation de patients ne reprenant pas une activité physique suffisante.

### 2.1 Résumé des bénéfices, le cas échéant, et des risques prévisibles et connus pour les personnes se prêtant à la recherche

L'implication et la surveillance mise en œuvre dans l'étude est un facteur de motivation quant à la reprise d'une activité physique.

### 2.2 Conformité de la recherche

La recherche sera conduite conformément aux principes de la dernière révision de la Déclaration d'Helsinki, au protocole, aux bonnes pratiques cliniques et aux dispositions législatives et réglementaires en vigueur.

### 2.3 Description de la population à étudier

Les patients recrutés pour cette étude sont des patients post chirurgie cardiaque au moment où ils quittent le secteur hospitalier.

### 2.4 Références à la littérature scientifique et aux données pertinentes servant de référence pour la recherche


1. Ball L, Costantino F, Pelosi P. Postoperative complications of patients undergoing cardiac surgery. Curr Opin Crit Care. 2016 Aug;22(4):386-92.

2. Kim DH, Kim CA, Placide S, et al. Preoperative Frailty Assessment and Outcomes at 6 Months or Later in Older Adults Undergoing Cardiac Surgical Procedures: A Systematic Review. Ann Intern Med 2016.

3. Mazzeffi M, Zivot J, Buchman T, Halkos M. In-hospital mortality after cardiac surgery: patient characteristics, timing, and association with postoperative length of intensive care unit and hospital stay. Ann Thorac Surg 2014; 97:1220.

4. Hulzebos EH1, Smit Y, Helders PP, van Meeteren NL. Preoperative physical therapy for elective cardiac surgery patients. Cochrane Database Syst Rev. 2012 Nov 14

5. Mainini C, Rebelo PF, Bardelli R, et al. Perioperative physical exercise interventions for patients undergoing lung cancer surgery: What is the evidence? SAGE Open Med. 2016 Oct 19

## 3. OBJECTIFS DE LA RECHERCHE, CRITERES EN RAPPORT AVEC CES OBJECTIFS

### 3.1. Objectif principal : Mesure

L'objectif principal de l'étude est de mesurer la reprise d'une activité physique après une chirurgie cardiaque programmée grâce à l'utilisation d'un bracelet électronique connecté. Cet objectif sera quantifié par le nombre de pas journaliers.

### 3.2. Objectifs secondaires

#### 3.2.1. Objectif secondaire : facteurs en rapport avec l'utilisation

Déterminer s'il existe un lien entre l'utilisation du bracelet et des critères pré-, per- et post-opératoires.

#### 3.2.2 Objectif secondaire 2 : facteur prédictifs de la reprise d'activité

Déterminer les critères pré-, per- et post-opératoires précoces (phase hospitalière) prédictifs d'une reprise d'activité physique conforme aux objectifs habituels fixés au patient avant sa sortie de l'établissement.

#### 3.2.3 Objectif secondaire 3 : lien avec la morbi−mortalité

Déterminer si la morbi−mortalité extra−hospitalière peut être prédite à à partir des données de surveillance de l'activité physique ainsi enregistrée.

## 4. CONCEPTION ET DEROULEMENT DE LA RECHERCHE

### 4.1. Plan expérimental

Il s'agit d'une étude prospective, ouverte et monocentrique effectuée à la sortie du secteur hospitalier des patients

### 4.2. Identification des sujets

Chaque sujet approché est identifié par l'initiale de son nom et de son prénom ainsi que par son numéro d'ordre de contact en vue de la recherche.

### 4.3. Déroulement de la recherche

Au moins 48 heures avant sa sortie du secteur hospitalier, chaque patient répondant aux critères d'éligibilité se voit proposer par un investigateur ou un médecin mandaté par celui−ci de participer à la recherche dont les modalités lui sont exposées verbalement, notemment le port d'un bracelet électronique.

Tout patient approché est identifié dans la première ligne disponible du registre des patients contactés (RPC) ce qui lui attribue automatiquement un numéro d'ordre.

Il est demandé aux patients qui se déclarent non intéressés d'indiquer sommairement leur raison qui est enregistrée dans le RPC ; les patients intéressés reçoivent une notice d'information.

Après au moins 12 heures de réflexion, le patient est approché et il lui est demandé de signer son consentement de participation. S'il ne souhaite pas participer, ses raisons pour ne pas participer seron recueillies et enregistrées dans le RPC.

Les patients qui acceptent de participer signent un consentement conforme à la réglementation en vigueur.

L'investigateur ou une personne qu'il mandate à cet effet installe une application (Medireport®) sur le téléphone portable ou la tablette du patient et expliquera à celui–ci où et comment porter le dispositif ainsi que la manière d'effectueront les transferts de données.

L'investigateur fournit au patient le mot de passe qui lui permettra ce transfert ; il connaît le mot de passe et peut donc à tout moment interroger la base de données pour prendre connaissance des données enregistrées.

Le bracelet électronique est porté en permanence par le patient à son poignet pendant toute la journée, c'est-à-dire du lever au coucher, entre le jour de la sortie du secteur hospitalier (J0) et la fin du deuxième mois extrahospitalier (J60). Les données enregistrées par le bracelet incluent : l'ID du bracelet, la date/heure, le nombre de pas effectué par journée, et la fréquence cardiaque moyennée par 5 minutes. Le bracelet connecté est relié à une base anonyme de données cloud permettant uniquement l'identification du bracelet. L'identification unique du dispositif sera enregistrée et liée au patient dans le dossier patient. Les données sont transférées toutes les heures par l'intermédiaire d'une application sur téléphone portable ou une tablette connectée à internet, pendant toute la durée de l'étude c'est-à-dire de la sortie du secteur hospitalier (J0) jusqu'au 60ème jour post-opératoire (J60). L'autonomie du dispositif est de plusieurs mois ce qui est donc suffisant pour la durée de l'étude (J0-J60).

Les données d'hospitalisation sont récupérées et directement reportées dans un eCRF.

Deux appel téléphonique à J30 et J60 seront réalisées par un technicien d'étude clinique mandaté par l'investigateur ; chaque appel utilisera un questionnaire structuré pour détecter la survenue d'évènements (cf. annexe 2-4) et obtenir des données sur le caractère pratique du dispositif. En cas d'inactivité du dispositif, le patient sera contacté par l'équipe en charge de l'étude pour en déterminer les raisons.

## 4.4. Tableau récapitulatif de la chronologie de la recherche

| **Actions** | **Visite d'inclusion** | **J30 après la sortie hospitalière** | **J60 après la sortie hospitalière** |
|---|---|---|---|
| Information | x | | |
| Recueil du consentement | x | | |
| Recueil des données hospitalières<br>Pré, per et post opératoire. | x | | |
| Formation du patient à l'utilisation du dispositif | x | | |
| Recueil des évènements extra hospitaliers sur appel téléphonique (cf. annexe 2) | | x | x |

| | | | |
|---|---|---|---|
| Questionnaire (cf. annexe 3) sur smartphone ou tablette via l'application ou appel téléphonique si pas de réponse. | | x | x |
| Questionnaire qualité de vie (cf. annexe 4) sur smartphone ou tablette via l'application ou appel téléphonique si pas de réponse. | | | x |

## 4.5. Description des techniques utilisées

Le bracelet électronique connecté utilisé dans cette étude est le « Withings Go » (cf.annexe 5), commercialisé dans tous les magasins spécialisés ainsi que l'application Medireport®. Il s'agit d'un bracelet léger de la taille d'une petite montre (cf. image ci-dessous).

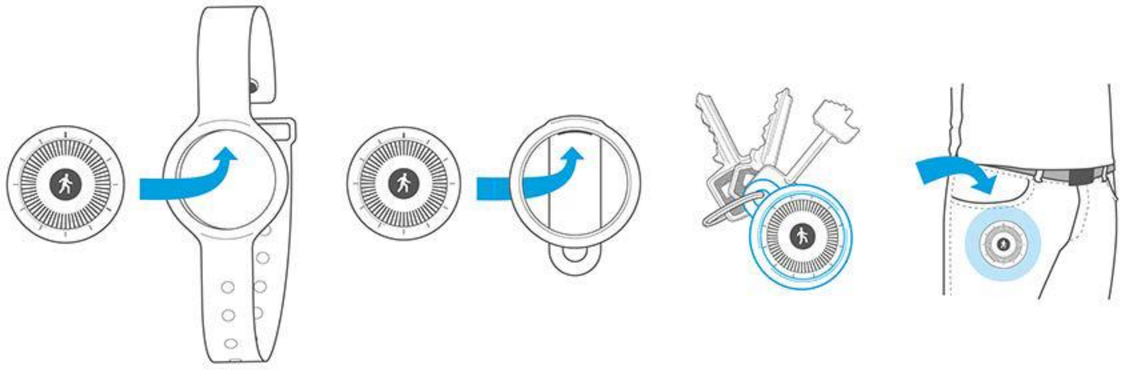

Un appel téléphonique sera réalisé à J30 et J60 de la sortie hospitalière avec questionnaire structuré portant sur la santé générale, l'activité physique, l'essoufflement, une éventuelle réhospitalisation, des douleurs, en particulier au niveau de la cicatrice, la qualité de vie et l'impact sur la vie du patient et l'utilité du bracelet connecté.

## 4.6. Durée prévue de participation des personnes, description de la chronologie et de la durée de la recherche

L'étude proprement dite débute au moment de la signature du consentement par le patient et l'investigateur. Elle se termine au 60$^{ème}$ jour après la sortie hospitalière.
Une période de 14 mois est prévue au maximum pour la réalisation de la recherche à partir de la date de la première inclusion.

## 4.7. Description des mesures prises pour réduire et éviter les biais

Un RPC est tenu à jour pour chaque patient contacté ; il indique si le patient a participé ou non à la recherche et vise à minimiser les biais de sélection.

## 4.8. Description des règles d'arrêt définitif ou temporaire

**De la participation d'une personne à la recherche**

Tout sujet peut arrêter sa participation à la recherche, à n'importe quel moment et quelle qu'en soit la raison. En cas d'arrêt prématuré de la recherche d'un sujet, les données déjà recueillies le concernant pourront être utilisées.

L'investigateur peut mettre fin à la participation de recherche d'un sujet, par exemple, pour non adhérence au protocole ou s'il considère que la poursuite de sa participation n'est pas optimale pour sa santé physique ou mentale.

Sauf urgence, il recueillera préalablement l'avis du gestionnaire de l'étude, et chaque fois que possible avertira préalablement le participant afin d'obtenir de celui-ci qu'il amende son comportement.

En cas d'interruption prématurée de la recherche pour un patient avant J30, celui-ci sera remplacé .

### 4.9. Identification de toutes les données à recueillir directement dans les cahiers d'observation, qui seront considérées comme des données source

Les données obtenues lors des appels téléphoniques à J30 et J60 par un professionnel de santé sont reportées directement dans l'eCRF (annexes 2-4).

En ce qui concerne les événements indésirables, on recueille pour tous les événements, la date de survenue, la nature de l'événements, la sévérité, l'évolution.

Les événements indésirables graves sont signalés au gestionnaire et conduisent comme prescrit par la réglementation à une enquête de vigilance dont les données serviront de données−sources.

## 5. POPULATION ETUDIEE

### 5.1. Description de la population à étudier

La population à étudier correspond aux patients majeurs, capable de comprendre et adhérant au projet de la recherche, au moment où ils quittent le secteur hospitalier après une intervention de chirurgie cardiaque.

### 5.2. Critères d'inclusion

Hommes ou femmes âgés de plus de 18 ans
ayant bénéficié d'une chirurgie cardiaque programmée, quelle que soit l'indication et le type d'intervention.
- donnant leur consentement de participation à l'étude.
- bénéficiant d'un régime de sécurité sociale

### 5.3. Critères de non-inclusion

- Refus du patient,
- Mauvaise compréhension du système (bracelet électronique, application) ou du principe de l'étude (problème de langue, séquelles vasculaires cérébrales),
- Handicap préexistant ne permettant pas la marche (non lié à la pathologie cardiaque ayant conduit à la chirurgie cardiaque programmée).
- Patient en incapacité de comprendre le contenu des informations délivrées.
- Femme enceinte.

- Patient qui ne disposent pas d'un téléphone intelligent ou qui ne peuvent se connecter à l'application ou dont on peut prévoir qu'ils ne pourront être contactés à J30 et/ou J60.

### 5.4. Critères d'exclusion et modalités

En cas d'inactivité du dispositif le patient sera contacté par l'équipe en charge de l'étude pour en comprendre la raison. Les patients n'adhérant pas au protocole ou refusant de porter le bracelet seront exclus et remplacés dans l'étude si l'exclusion intervient avant J30.

### 5.5. Modalités de recrutement

L'avant–veille de la sortie hospitalière tous les patients ayant eu une intervention de chirurgie cardiaque se verront proposer de participer à l'étude.

## 6. EVALUATION DE LA SECURITE

Dans le cadre des recherches visant à évaluer les soins courants, les actes ou les stratégies médicales, objets de la recherche, font partie de la pratique habituelle et sont utilisés dans le respect de leurs indications. Les événements indésirables potentiels sont donc ceux liés à la prise en charge habituelle du patient (liés aux soins) et ne requièrent pas de déclaration spécifique de la part du gestionnaire de la recherche.

Dans le cadre de cette étude, la survenue d'évènements indésirables sera néanmoins recueillie et reportés dans l'eCRF lors des appels téléphoniques à J30 et J60 ou pourra directement être signalée par les patients sur l'application Medireport dédiée au suivi des patients dans l'étude.

## 7. STATISTIQUES

Un plan d'analyse statistique résumé ci–dessous sera finalisé une fois que le protocole aura été autorisé par les autorités compétentes et aura fait l'objet d'un avis favorable du CPP.
Ce plan fera l'objet d'un document écrit soumis au gestionnaire de l'étude.

### 7.1. Description des méthodes statistiques prévues

Il s'agit essentiellement d'une analyse descriptive à laquelle on pourra éventuellement ajouter un temps exploratoire. Pour les variables catégorielles, l'effecif global et l'effectif dans chaque catégorie ainsi que les proportions correspondantes et leurs limites de confiance seront fournies. Pour les variables continues, la moyenne et son écart–type, la médiane et les limites du premier et dernier quartiles, l'effectif disponible seront fournis. Les variables ordinales seront traitées à la fois comme des variables catégorielles (effectif et pourcentages) et comme des variables continues. Dans le temps exploratoire de l'analyse, on cherchera à rattacher les données concernant les variables dépendantes (activité, usage, morbi–mortalité aux variables indépendantes recueillies en pré–, per– et post–opératoire par des analyses bivariées (coefficient de corrélation) ou des analyses multivariées (régression multiple, régression logistique ou analyse des correspondances).

### 7.2. Nombre prévu de personnes à inclure dans la recherche avec sa justification statistique

Il s'agit d'une étude exploratoire ; ce qui rend difficile le calcul du nombre de sujets à inclure.
En se basant sur les capacités de recrutement, l'objectif de conclure rapidement quant à l'intérêt de la méthode de manière à pouvoir, le cas échéant, mener une étude comparative, et la nécessité de disposer d'une puissance suffisante. Un échatillon de 100 patients permet, si une proportion est comprise entre 0.1 et 0.5 d'obtenir des limites de confiance à 95 % d'une taille raisonnable.

| Limites de confiance | (N) | 2.4.1.1 Taille de l'intervalle | (P) | Limite. inférieure | 2.4.1.2 Limite supérieure |
|---|---|---|---|---|---|
| 0.950 | 100 | 0.127 | 0.100 | 0.049 | 0.176 |
| 0.950 | 100 | 0.149 | 0.150 | 0.086 | 0.235 |
| 0.950 | 100 | 0.165 | 0.200 | 0.127 | 0.292 |
| 0.950 | 100 | 0.178 | 0.250 | 0.169 | 0.347 |
| 0.950 | 100 | 0.187 | 0.300 | 0.212 | 0.400 |
| 0.950 | 100 | 0.195 | 0.350 | 0.257 | 0.452 |
| 0.950 | 100 | 0.199 | 0.400 | 0.303 | 0.503 |
| 0.950 | 100 | 0.202 | 0.450 | 0.350 | 0.553 |
| 0.950 | 100 | 0.203 | 0.500 | 0.398 | 0.602 |

On approchera donc le nomre de patients nécessair pour obtenir 100 consentements de participation.

### 7.3. Degré de signification prévu

Les limites de confiance à 95 % seront données pour les variables dont on fera l'analyse descrpitive.

### 7.4. Critères statistiques d'arrêt de la recherche

Il n'est pas prévu d'analyse intermédiaire.

### 7.5. Méthode de prise en compte des données manquantes, inutilisées ou non valides

Les données recueillies seront soumises à un comité de validation. Il n'est pas prévu de méthode particulière d'imputation des données manquantes.

### 7.6. Gestion des modifications apportées au plan statistique initial

Les modifications au plan d'analyse statistique feront l'objet d'un document écrit soumis au gestionnaire. Si ces modifications interviennent après la réunion de validation et le verrouillage de la base de données, les résultats qui en résulteraient seraient considérés comme provenant d'analyses post–hoc dont les niveaux de probabilité ne sont qu'indicatifs.

### 7.7. Choix des personnes à inclure dans les analyses

Tous les patients figurant dans le RPC seront pris en compte pour décrire l'évolution de l'échantillon de population concerné par la recherche et en particulier les refus de participation et leurs causes.
Les données de tous les patients ayant donné leur consentement de participation seront utilisées pour toutes les autres analsyes.

## 8. DROIT D'ACCES AUX DONNEES ET DOCUMENTS SOURCE

### 8.1. Accès aux données

Le gestionnaire de l’étude a obtenu l’accord de l’ensemble des parties impliquées dans la recherche afin de garantir l’accès direct à tous les lieux de déroulement de la recherche, aux

données sources, aux documents sources et aux rapports dans un but de contrôle de qualité et d'audit par le gestionnaire et/ou les autorités compétentes.
Les investigateurs mettront les documents et données individuelles strictement nécessaires au suivi, au contrôle de qualité et à l'audit de la recherche biomédicale, à la disposition des personnes mandatées par le gestionnaire conformément aux dispositions législatives et réglementaires en vigueur (articles L.1121-3 et R.5121-13 du code de la santé publique).

## 8.2. DOCUMENTS SOURCE

Les documents source sont définis comme les documents ou objets originaux permettant de prouver l'existence ou l'exactitude d'une donnée ou d'un fait enregistrés au cours de la recherche. Ils seront conservés pendant 15 ans par l'investigateur ou par l'hôpital s'il s'agit d'un dossier médical hospitalier.

## 8.3. CONFIDENTIALITE DES DONNEES

Conformément aux dispositions concernant la confidentialité des données auxquelles ont accès les personnes chargées du contrôle de qualité d'une recherche biomédicale (articles L.1121-3 et R.5121-13 du code de la santé publique), les personnes ayant un accès direct prendront toutes les précautions nécessaires en vue d'assurer la confidentialité des informations relatives aux personnes qui se sont prêtées à la recherche notamment en ce qui concerne leur identité ainsi qu'aux résultats obtenus. Ces personnes, au même titre que les investigateurs eux-mêmes, sont soumises au secret professionnel (selon les conditions définies par les articles 226-13 et 226-14 du code pénal).
Pendant la recherche ou à son issue, les données recueillies sur les personnes qui s'y prêtent et transmises au gestionnaire de l'étude par les investigateurs (ou tous autres intervenants spécialisés) seront rendues non identifiants.
Elles ne doivent en aucun cas faire apparaître en clair les noms des personnes concernées ni leur adresse.
Ces personnes ne seront connues que par leur numéro patient : n° ordre d'inclusion de la personne dans le centre (3 positions numériques) - initiale nom - initiale prénom.
Cette référence sera unique et sera conservée pour toute la durée de la recherche. Ces éléments sont présents dans le cahier d'observation. Les données recueillies sont strictement confidentielles. Elles ne sont consultées que par l'équipe médicale, les personnes dûment mandatées par le gestionnaire de la recherche et éventuellement par des représentants des autorités sanitaires et judiciaires habilitées. L'identité des participants ne sera révélée dans aucun rapport ou publication résultant de cette étude.
Le gestionnaire de l'étude s'assurera que chaque personne qui se prête à la recherche a donné son accord par écrit pour l'accès aux données individuelles la concernant et strictement nécessaires au contrôle de qualité de la recherche.

## 8.4. CAHIER D'OBSERVATION ET RECUEIL DES DONNEES

Toutes les informations requises par le protocole doivent être consignées sur les Cahiers de Recueil Formalisés (CRF ou e–CRF) spécifiques à l'étude. Le recueil des données est réalisé par l'investigateur et/ou le personnel désigné par l'investigateur.
Les données devront être recueillies au fur et à mesure qu'elles sont obtenues, et transcrites dans ces cahiers de façon nette et lisible.
Les données concernant les sujets, collectées sur les cahiers d'observation pendant l'essai, le seront de façon anonyme et le sujet ne sera identifié que par son numéro patient.
La base de données pour la saisie, la gestion et l'analyse des données des cahiers d'observation papier sera développée, maintenue et hébergée par le CMC Ambroise Paré ou par un prestataire mandaté par ce dernier. La saisie des données collectées dans l'eCRF sera réalisée de manière à strictement respecter la confidentialité et les patients ne seront identifiés que par un code alphanumérique.
Les données informatisées seront recueillies conformément à la méthodologie MR–001 de la CNIL.

## 9. CONTROLE ET ASSURANCE DE LA QUALITE

### 9.1. CONTRÔLE QUALITE DES DONNEES

La recherche sera encadrée selon les procédures opératoires standards du gestionnaire.
Le déroulement de la recherche et la prise en charge des sujets se feront conformément à la réglementation et les Bonnes Pratiques en vigueur.

### 9.2. AUDIT/INSPECTION

Les auditeurs devront avoir un accès direct aux données sources et médicales et à tout document utile lié à la conduite de l'étude clinique.
La confidentialité des données et l'anonymat des patients seront alors respectés.
La personne qui dirige et surveille la recherche accepte de se conformer aux exigences du promteur en ce qui concerne un audit de la recherche.

## 10 CONSIDERATIONS ETHIQUES

### 10.1. JUSTIFICATION DE LA TYPOLOGIE DE LA RECHERCHE EN SOINS COURANTS

Compte tenu de l'ensemble de ces éléments, le responsable de la recherche qualifie celle-ci en première intention de **recherche en soins courants**, puisque :
tous les actes sont pratiqués et les produits utilisés de manière habituelle
les modalités particulières de mises en œuvre dans la recherche doivent diminuer les risques et représenter des contraintes négligeables pour la personne qui se prête à la recherche. (Article R 1121-3 du code de la santé publique (CSP), décret n° 2006-477 du 26 avril 2006).

### 10.2. DEMARCHES REGLEMENTAIRES PREALABLES A LA MISE EN ŒUVRE DE LA RECHERCHE

#### 10.2.1. ANSM

#### 10.2.2. Comité de Protection des Personnes

Cette recherche n'entre pas dans le cadre de la loi du 9 août 2004 car les actes pratiqués et/ou les produits utilisés le sont de manière habituelle sans procédures invasives supplémentaires ou inhabituelles de diagnostic ou de surveillance
.
Le gestionnaire de la recherche, soumettra, avant toute mise en œuvre de la recherche, pour avis et confirmation de la qualification de la recherche, le protocole au Comité de Protection des Personnes, conformément à l'article L 1121-1 du code de la santé publique (CSP) tels qu'ils résultent des lois n° 2004-806 du 9 août 2004 et n° 2006-450 du 18 avril 2006 relative à la politique de santé publique.

Le CPP concerné est celui d'Ile de France VII, Hôpital Bicêtre. L'avis du comité mentionné ci-dessus sera notifié dans la note d'information donnée aux personnes concernées.

#### 10.2.2. CNIL

Cette recherche est soumise à la loi n°78-17 du 6 janvier 1978 modifiée par la loi n° 2004-801 du 6 août 2004 relative à la protection des personnes physiques à l'égard des traitements de données à caractère personnel.

Cette recherche, monocentrique, fera l'objet d'une déclaration normale auprès de la CNIL par le gestionnaire de l'étude
Les informations relatives aux droits des personnes participant à cette recherche sont intégrées dans la note d'information.

## 11. TRAITEMENT DES DONNEES ET CONSERVATION DES DOCUMENTS ET DES DONNEES RELATIVES A LA RECHERCHE

Les documents spécifiques de la recherche devront être archivés, par toutes les parties, sous le nom de l'étude dans les locaux attribués à cet effet jusqu'à la fin de la période d'utilité pratique (15 ans après la fin de la recherche).
Ces documents sont :
- Le protocole et annexes spécifiques de l'étude, les amendements éventuels,
- L'autorisation de l'ANSM, les avis du CPP et la déclaration normale auprès de la CNIL,
- Les formulaires d'information et consentements signés (sous enveloppes scellées pour le gestionnaire),
- La liste des inclusions (RPC),
- Les cahiers d'observation complétés et validés de chaque sujet inclus,
- Les documents éventuels de suivi,
- Les analyses statistiques,
- Le rapport final de l'étude,
- Les certificats d'audit éventuellement réalisés au cours de la recherche.

Aucun déplacement ou destruction ne pourra être effectué sans l'accord du gestionnaire de l'étude et/ou de l'investigateur. Toutes les données, tous les documents et rapports pourront faire l'objet d'audit.

## 12. FINANCEMENT ET ASSURANCE

### FINANCEMENT DE L'ETUDE

Il est assuré par le gestionnaire

### ASSURANCE

Dans la mesure où il s'agit d'un soin courant sans risque spécifique identifié, l'assurance sera couverte par le contrat habituel. Cette assurance couvre la responsabilité du gestionnaire et des autres intervenants et peut être consultée à la Direction du CMC Ambroise Paré.

## 13. REGLES RELATIVES A LA PUBLICATION

La recherche sera enregistrée sur le site Clinical Trials.

Cette étude fera l'objet de publications sous formes de communication et d'article original.
L'ordre des signataires sera précisé ultérieurement.

## 14. REFERENCE

1. Ball L, Costantino F, Pelosi P. Postoperative complications of patients undergoing cardiac surgery. Curr Opin Crit Care. 2016 Aug;22(4):386-92.

2. Kim DH, Kim CA, Placide S, et al. Preoperative Frailty Assessment and Outcomes at 6 Months or Later in Older Adults Undergoing Cardiac Surgical Procedures: A Systematic Review. Ann Intern Med 2016.

3. Mazzeffi M, Zivot J, Buchman T, Halkos M. In-hospital mortality after cardiac surgery: patient characteristics, timing, and association with postoperative length of intensive care unit and hospital stay. Ann Thorac Surg 2014; 97:1220.

4. Hulzebos EH1, Smit Y, Helders PP, van Meeteren NL. Preoperative physical therapy for elective cardiac surgery patients. Cochrane Database Syst Rev. 2012 Nov 14

5. Mainini C, Rebelo PF, Bardelli R, et al. Perioperative physical exercise interventions for patients undergoing lung cancer surgery: What is the evidence? SAGE Open Med. 2016 Oct 19

The Kansas City cardiomyopathy questionnaire: JACC vol 35, No 5, 2000. April 2000: 1245-55

## 15. ANNEXES

**Annexe 1** Recueil de données intrahospitalières

Données pré-opératoires

- Patient ID
- Date de Naissance, Age
- Sexe,
- Poids, Taille, IMC
- Antécédents coronariens : infarctus, angioplastie, pontages
- Antécédents non-coronarien : AVC, pathologie vasculaire périphérique, pacemaker, fibrillation atriale, anticoagulation, valvulopathie, BPCO, immunodépression, toute cause de handicap gênant la marche
- Facteur de risque CV : HTA, Tabac, Diabète, Hypercholestérolémie
- Angor
- Insuffisance cardiaque : stade NYHA
- Echocardiographie : FEVG, PAPs
- Créatininémie
- Score STS
- Euroscore I et II
- Score Syntax

Données opératoires

- Date intervention
- Type d'intervention
- Durée de la CEC
- Complications per-opératoires
- Date de sortie de réanimation

Données post-opératoires hospitalières

- Complications hospitalières :

o Date
o Description
o Sévérité mineure ou majeure
o Prolongation de la durée de séjour
o Lien avec l'intervention
o Décès : cause
- Date de sortie de l'Hôpital (J0)

**Annexe 2** Recueil de données extra-hospitalières

Suivi à 1 mois (J30) par téléphone
Evènements extra-hospitaliers :
o Date
o Description
o Sévérité mineure ou majeure
o Prolongation de la durée de séjour ? rehospitalisation
o Lien avec l'intervention
o Décès : cause

Suivi à 2 mois (J60) par téléphone
Evènements extra-hospitaliers :
o Date
o Description
o Sévérité mineure ou majeure
o Prolongation de la durée de séjour
o Lien avec l'intervention
o Décès : cause

**Annexe 3**

Questionnaire patient utilisé pour l'appel téléphonique et/ou adressé par smartphone.

Comment estimez-vous votre activité physique depuis votre intervention :
o Identique
o Améliorée
o Moins bonne
Comment estimez-vous votre essoufflement sur une échelle de 0 à 10 ???
o Identique
o Améliorée
o Moins bonne
Avez-vous été ré hospitalisé depuis la sortie de la clinque Ambroise Paré
o Oui
o Non
Ressentez-vous des douleurs au niveau de votre cicatrice
o Oui
o Non
Si Oui , ces douleurs limitent elle la reprise de votre activité
o Oui
o Non

Pensez-vous que le port du bracelet connecté a pu influencer votre reprise d'activité
o Oui
o Non
o Ne sais pas
Le bracelet connecté a-t-il représenté une contrainte dans votre vie quotidienne ?

o Oui
o Non
Ce type de surveillance a-t-il changé votre mode de vie ?
o Oui
o Non
Accepteriez-vous de poursuivre votre surveillance médicale avec ce même bracelet
o Oui
o Non
Pensez-vous qu'un autre objet connecté (pèse personne, montre, téléphone …) permettrait d'améliorer votre prise en charge et surveillance médicales à long terme.

Annexe 4

# QUALITE DE VIE AUJOURD'HUI
## EUROQOL : EQ-5D

**N° patient : |__|__|__|__|__|__| Initiales : |__|__|**

Pour chaque rubrique, veuillez cocher UNE case, celle qui décrit le mieux votre santé aujourd'hui.

| | |
|---|---|
| **QL01 Mobilité** | |
| 1. Je n'ai aucun problème pour me déplacer à pied. | ❑ |
| 2. J'ai des problèmes légers pour me déplacer à pied. | ❑ |
| 3. J'ai des problèmes modérés pour me déplacer à pied. | ❑ |
| 4. J'ai des problèmes sévères pour me déplacer à pied. | ❑ |
| 5. Je suis incapable de me déplacer à pied. | ❑ |
| **QL02 Autonomie de la personne** | |
| 1. Je n'ai aucun problème pour me laver ou m'habiller tout(e) seul(e). | ❑ |
| 2. J'ai des problèmes légers pour me laver ou m'habiller tout(e) seul(e). | ❑ |
| 3. J'ai des problèmes modérés pour me laver ou m'habiller tout(e) seul(e). | ❑ |
| 4. J'ai des problèmes sévères pour me laver ou m'habiller tout(e) seul(e). | ❑ |
| 5. Je suis incapable de me laver ou de m'habiller tout(e) seul(e). | ❑ |
| **QL03 Activités courantes (exemples : travail, études, travaux ménagers, activités familiales ou loisirs)** | |
| 1. Je n'ai aucun problème pour accomplir mes activités courantes. | ❑ |
| 2. J'ai des problèmes légers pour accomplir mes activités courantes. | ❑ |
| 3. J'ai des problèmes modérés pour accomplir mes activités courantes. | ❑ |
| 4. J'ai des problèmes sévères pour accomplir mes activités courantes. | ❑ |
| 5. Je suis incapable d'accomplir mes activités courantes. | ❑ |
| **QL04 Douleurs / gêne** | |
| 1. Je n'ai ni douleur ni gêne. | ❑ |
| 2. J'ai des douleurs ou une gêne légère(s). | ❑ |
| 3. J'ai des douleurs ou une gêne modérée(s). | ❑ |
| 4. J'ai des douleurs ou une gêne sévère(s). | ❑ |
| 5. J'ai des douleurs ou une gêne extrême(s). | ❑ |
| **QL05 Anxiété / dépression** | |
| 1. Je ne suis ni anxieux(se), ni déprimé(e). | ❑ |
| 2. Je suis légèrement anxieux(se) ou déprimé(e). | ❑ |
| 3. Je suis modérément anxieux(se) ou déprimé(e) | ❑ |
| 4. Je suis sévèrement anxieux(se) ou déprimé(e). | ❑ |
| 5. Je suis extrêmement anxieux(se) ou déprimé(e). | ❑ |

A présent, indiquez sur l’échelle ci-dessous à combien vous estimez votre état de santé aujourd’hui entre 0 et 100, sachant que 100 correspond au meilleur état de santé et 0 au pire état de santé que vous puissiez imaginer.

| Pire Etat de santé imaginable | Meilleur Etat de santé imaginable |
|---|---|

I__I__I__I__I__I__I__I__I__I__I__I__I__I__I__I__I__I__I__I__I

0 10 20 30 40 50 60 70 80 90 100

Votre état de santé aujourd’hui = [ ]

**Annexe 5**

La notice du bracelet électronique connecté de la marque Withings Go est jointe à part.

Pierre SQUARA le 7 novembre 2016

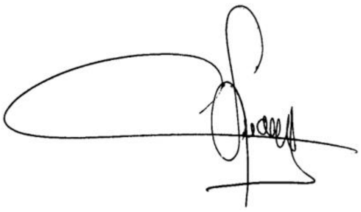

Supplement: S4 File — (PDF) [file pone.0241368.s004.pdf]
